# Oesophageal Soft Food Bolus Obstruction: A Retrospective Review of the Management of 384 Cases in Two UK Hospitals

**DOI:** 10.7759/cureus.99702

**Published:** 2025-12-20

**Authors:** Faizan Shah, Aimee Love, Fergus Cooper, Hayfa Sheikh, Douglas Naismith, Muhammad Shakeel

**Affiliations:** 1 Otolaryngology - Head and Neck Surgery, Aberdeen Royal Infirmary, Aberdeen, GBR; 2 School of Medicine and Dentistry, University of Aberdeen, Aberdeen, GBR; 3 General Practice, University of Aberdeen, Aberdeen, GBR

**Keywords:** deliberate foreign body ingestion, esophageal foreign body, food bolus, foreign body ingestion treatment, oesophageal soft food bolus obstruction, swallowed foreign body

## Abstract

Objectives

Oesophageal soft food bolus obstruction (OSFBO) is a frequent emergency presentation to otolaryngology, gastroenterology, and general surgery departments. While many cases resolve without intervention, others require urgent endoscopy, with no UK consensus on referral pathways, timing, or technique. This study evaluates the real-world management of OSFBO across two UK hospitals over 12 years, examining rates of conservative resolution, procedural intervention, associated complications, and the diagnostic utility of post-resolution contrast swallow imaging.

Methods

A retrospective review of all adult patients (≥16 years) presenting with OSFBO between August 2008 and August 2020 at one tertiary and one district general hospital was conducted. Sharp or non-organic foreign body cases were excluded. Data were collected from electronic records, anonymized, and analysed using IBM SPSS (IBM Corp., Armonk, NY). Categorical and continuous variables were compared using appropriate statistical tests. Kaplan-Meier survival analysis assessed time to resolution, and logistic regression (visualized via a forest plot) identified predictors of procedural intervention.

Results

Among 384 cases, 65% (n = 249) resolved with conservative management. Of the 135 patients requiring intervention, 58% (n = 70) underwent rigid oesophagoscopy, and 42% (n = 51) had flexible oesophagoscopy. One case of oesophageal perforation occurred following rigid oesophagoscopy (1.4% complication rate). Flexible nasendoscopy dislodged the bolus in 3% (n = 7) of all cases. Hyoscine butylbromide was used in 71% (n = 193), with 32% (n = 62) subsequently requiring intervention, compared to 6% (n = 5) in those not receiving it. Plain radiographs were diagnostic in only 6% (n = 7) of 122 cases. Post-resolution contrast swallow imaging was performed in 20% (n = 75), with 48% (n = 36) revealing oesophageal abnormalities such as webs, achalasia, and strictures. Kaplan-Meier analysis showed most conservative resolutions occurred within 24-48 hours. Regression analysis identified age and absence of hyoscine as significant predictors of intervention.

Conclusions

The majority of OSFBO cases resolve without the need for invasive procedures. Rigid and flexible oesophagoscopy are both effective, though rigid carries a higher risk of complications. Flexible nasendoscopy may offer a low-risk, bedside option for select patients. Post-resolution contrast swallow studies have a high diagnostic yield and should be considered in follow-up. These findings support the need for national guidelines and further prospective research into pharmacological and procedural strategies for OSFBO.

## Introduction

Oesophageal soft food bolus obstruction (OSFBO) is a common acute presentation, typically characterized by dysphagia, regurgitation, and significant discomfort or distress for the patient [1]. If left untreated, OSFBO can result in serious morbidity, including dehydration, aspiration pneumonia, oesophagitis, oesophageal perforation, mediastinitis, and, in severe cases, death [2]. In instances where a sharp or corrosive foreign body has been ingested, urgent operative intervention is required to minimize the risk of mucosal injury and perforation [3]. Imaging techniques, such as soft tissue neck radiography or computed tomography (CT), are generally reserved for cases where the nature of the ingested material is unclear or when there is concern that it may be sharp or radiopaque [4].

Patients with OSFBO may be referred either to gastroenterology or otolaryngology, depending on the site of obstruction and local hospital protocols. While there are established management guidelines from both American [4] and European [5] gastroenterological societies, there are currently no national otolaryngology guidelines within the United Kingdom for the management of this condition [6].

Treatment approaches for OSFBO include conservative management (such as watchful waiting), pharmacological therapies, and endoscopic intervention [2]. First-line pharmacological agents may include smooth muscle relaxants or antispasmodics. Gastroenterologists typically manage these cases with flexible oesophagoscopy under sedation, whereas otolaryngologists often employ rigid oesophagoscopy, which necessitates general anaesthesia [7,8]. Each modality carries its own risks and logistical considerations, and institutional preferences often determine the choice of procedure.

Many patients presenting with OSFBO are found to have underlying oesophageal abnormalities, including strictures, malignancies, or inflammatory conditions such as eosinophilic oesophagitis (EoE) [9]. EoE, in particular, is considered an underdiagnosed cause of food bolus obstruction, especially in younger adults. Some studies have reported a prevalence as high as 80% in this population [10,11]. However, EoE is also associated with increased fragility of the oesophageal mucosa, placing these patients at greater risk of complications such as perforation during endoscopic intervention [12].

Although spontaneous resolution of OSFBO may occur in some cases [13], prolonged obstruction can lead to mucosal trauma and inflammation, and delayed intervention may increase the likelihood of complications [3]. Therefore, timely and appropriate management is essential.

Due to the acute and unpredictable nature of this condition, high-quality randomized trials are difficult to conduct, and much of the available evidence is derived from retrospective case series [2]. As such, retrospective analyses remain a valuable resource in understanding current practice and informing management decisions. The aim of this study is to present a large retrospective case series of patients treated for OSFBO at two UK-based NHS hospitals. The findings are compared with similar reports in the literature, with the objective of contributing to the evidence base and informing clinical practice in otolaryngology departments.

## Materials and methods

Study design and setting

This retrospective observational cohort study analysed adult patients presenting with OSFBO at two NHS hospitals in the United Kingdom from August 1, 2008, to August 31, 2020. The participating centres comprised a tertiary referral hospital and a district general hospital, ensuring a diverse patient population across secondary and tertiary care settings. The study aimed to evaluate demographic characteristics, management strategies, including conservative and procedural interventions, complication rates, and outcomes in patients with OSFBO.

Inclusion and exclusion criteria

Patients aged 16 years or older presenting with clinical features consistent with OSFBO, such as acute dysphagia, inability to swallow saliva, or odynophagia following ingestion of solid food, were eligible for inclusion. Only cases involving organic food bolus impactions were considered. Exclusion criteria included foreign bodies with sharp or non-organic characteristics (e.g., fish or chicken bones, coins, batteries, and dentures), intentional ingestion related to psychiatric conditions, and recurrent presentations of the same patient during the study period to avoid duplication.

Data collection

The data were retrospectively extracted from electronic patient records (EPR) across both hospitals using a standardized template to ensure uniformity. Data sources included emergency department records, inpatient clinical notes, ENT and gastroenterology consultations, operative and procedural reports, imaging records, and discharge summaries.

Collected variables encompassed patient demographics (age, gender), referral source and timing, pharmacological management (use of hyoscine butylbromide), clinical outcomes including resolution without procedural intervention, and types of procedural interventions performed. Procedural interventions were categorized into rigid oesophagoscopy, flexible oesophagoscopy, and transnasal flexible oesophagoscopy (nasendoscopy), the latter being considered a procedural intervention despite its frequent use in ward-based settings. The nature of the obstructing food bolus was recorded where documented (meat, bread, mixed). Procedural complications, including oesophageal mucosal trauma, bleeding, aspiration, and perforation, were noted. Follow-up investigations with contrast swallow radiography and their findings were also recorded.

Certain variables were excluded due to inconsistent documentation, including the precise anatomical level of obstruction, use of alternative pharmacological agents such as glucagon, diazepam, or effervescent drinks, and long-term follow-up data beyond the index admission. Comorbidities and other patient medical histories were similarly not analysed due to limited availability.

Data management and statistical analysis

All patient data were anonymized and de-identified prior to analysis to maintain confidentiality and comply with data protection regulations. Data were collated in a secure database and analysed using IBM SPSS Statistics version 27 (IBM Corp., Armonk, NY).

Descriptive statistics summarized the cohort characteristics. Categorical data were reported as frequencies and percentages (N, %), while continuous variables were described using medians with interquartile ranges (IQR) due to non-normal distributions. Comparative analyses were performed using chi-square or Fisher’s exact tests for categorical variables, and Mann-Whitney U tests for continuous variables to assess differences between patients managed conservatively and those requiring procedural intervention. Statistical significance was set at p < 0.05.

Multivariate logistic regression analysis was employed to identify factors associated with the need for procedural intervention. Adjusted odds ratios (OR) with 95% confidence intervals (CI) were calculated and presented in a forest plot generated using SPSS.

Time-to-resolution analyses were conducted with Kaplan-Meier survival curves to illustrate differences in time to symptom resolution between management strategies. Log-rank tests compared survival distributions across groups. Kaplan-Meier curves were also generated using SPSS.

Ethical considerations and governance

The study was registered with the Clinical Effectiveness and Audit Departments at NHS Grampian before data collection commenced. Given the retrospective design using anonymized, routinely collected clinical data for service evaluation and quality improvement, formal ethical approval was deemed unnecessary per the UK Health Research Authority (HRA) guidelines. All procedures conformed to the UK General Data Protection Regulation (GDPR) and institutional data governance policies, ensuring no identifiable patient information was included in analysis or dissemination.

## Results

Patient demographics and presentation

A total of 384 patients met the inclusion criteria. Demographic details are summarized in Table 1. The median age at presentation was 53.5 years (interquartile range (IQR) = 21-83). Females presented at a significantly older age than males, with a median of 67.5 years (IQR = 23-87) versus 47.5 years (IQR = 20-77), respectively (Mann-Whitney U test, p = 0.01). Most patients (82%, n = 315) presented within 24 hours of symptom onset, and 92% (n = 353) within 48 hours. The majority were referred from the emergency department (75%, n = 288), with 24% (n = 92) referred from primary care.

**Table 1:** Demographic breakdown of patients presenting with OSFBO (n = 384). * Mann–Whitney U test comparing median age by gender. ** Chi-square test comparing referral source distribution. OSFBO: oesophageal soft food bolus obstruction; N/A: not applicable.

Characteristic	N (%)	Statistical analysis
Gender		
Male	238 (62.0)	N/A
Female	146 (38.0)	
Age at presentation (years)		Median age: 67.5 vs. 47.5 years (p = 0.01)*
16–24	62 (16.1)	
25–49	101 (26.3)	
50–64	73 (19.0)	
> 65	136 (35.4)	
Unknown	12 (3.1)	
Referral source		χ²(1) = 88.7, p < 0.001**
Emergency department	288 (75.0)	N/A
Primary care	92 (24.0)	

Characteristics of the food bolus and initial investigations

The nature of the food bolus was documented in 70% (n = 267) of cases, with meat comprising 83% (n = 221) of these obstructions. Flexible nasendoscopy was performed in 53% (n = 205) of patients; the food bolus was visualized in 5% (n = 11) and dislodged in 3% (n = 7) of cases using this technique. Radiological imaging (neck or chest X-rays) was performed in 32% (n = 122) of patients, detecting signs of impaction in 6% (n = 7).

Pharmacological management

Data on hyoscine butylbromide administration were available for 270 patients. Among these, 71% (n = 193) received at least one dose. Of those treated with hyoscine, 32% (n = 62) subsequently required procedural intervention, compared to 6% (n = 5) in the group not receiving the drug, a statistically significant difference (chi-square test, p < 0.001).

Management outcomes

The breakdown of OSFBO resolution methods is presented in Table 2. Conservative management without procedural intervention resolved 65% (n = 249) of cases. Rigid oesophagoscopy accounted for resolution in 18% (n = 70), flexible oesophagoscopy in 13% (n = 51), and flexible nasendoscopy/transnasal oesophagoscopy in 2% (n = 7). Procedural details were unknown in 2% (n = 7) of cases.

**Table 2:** Pathologies identified on contrast swallow (n = 36). * Other pathologies: silent laryngeal aspiration, oesophageal compression from external mass, oesophageal dilatation, and large vallecular cyst.

Pathology	N (%)
Oesophageal web	8 (22.2)
Achalasia	8 (22.2)
Gastro-oesophageal reflux	4 (11.1)
Oesophageal stricture	4 (11.1)
Oesophageal dysmotility	3 (8.3)
Hiatus hernia	3 (8.3)
Pharyngeal pouch	2 (5.6)
Other*	4 (11.1)

One complication occurred, i.e., a single oesophageal tear following rigid oesophagoscopy, which was managed conservatively with antibiotics and nasogastric feeding, prolonging the hospital stay. No complications were reported among conservatively managed patients.

Post-resolution imaging findings

Contrast swallow radiography was performed in 20% (n = 75) of patients after OSFBO resolution. Of these, 48% (n = 36) showed abnormalities (Table 3). The most frequent findings were oesophageal webs and achalasia, each identified in 22% (n = 8) of cases. Other detected abnormalities included gastro-oesophageal reflux (11%, n = 4), oesophageal strictures (11%, n = 4), oesophageal dysmotility (8%, n = 3), hiatus hernia (8%, n = 3), and pharyngeal pouch (6%, n = 2). Additional pathologies comprised silent laryngeal aspiration, external oesophageal compression, oesophageal dilatation, and vallecular cyst (11%, n = 4).

**Table 3:** Method of resolution of OSFBO (n = 384). OSFBO: oesophageal soft food bolus obstruction; N/A: not applicable.

Resolution method	N (%)	Notes
Resolved without procedural intervention	249 (64.8)	N/A
Resolved by rigid oesophagoscopy	70 (18.2)	
Resolved by flexible oesophagoscopy	51 (13.3)	
Resolved by flexible nasendoscopy/transnasal oesophagoscopy	7 (1.8)	
Resolved by procedural intervention (details unknown)	7 (1.8)	
Complications	1 (0.3)	Oesophageal tear after rigid oesophagoscopy

Factors associated with procedural intervention

**Figure 1:**
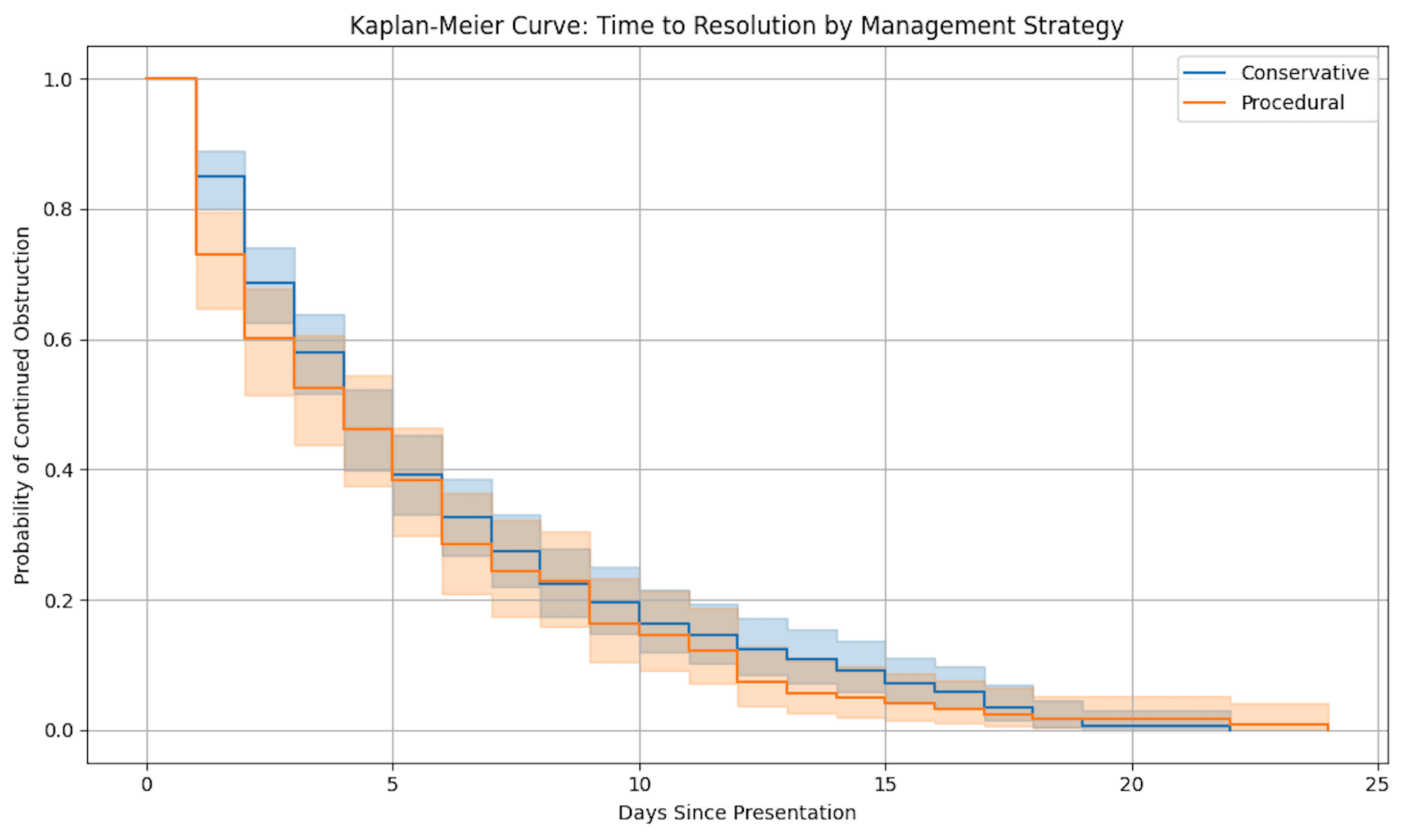
Kaplan-Meier curve demonstrating time to resolution of symptoms in patients with OSFBO by management strategy. This Kaplan-Meier survival curve illustrates the probability of symptom resolution over time among patients presenting with oesophageal soft food bolus obstruction (OSFBO). The curve compares patients managed conservatively versus those who underwent procedural intervention (rigid, flexible, or transnasal oesophagoscopy). Median time to resolution was significantly shorter in the procedural intervention group compared to the conservative management group (log-rank test, p < 0.001). The shaded areas represent 95% confidence intervals for each group. This analysis underscores the effectiveness of procedural interventions in accelerating symptom relief.

**Figure 2:**
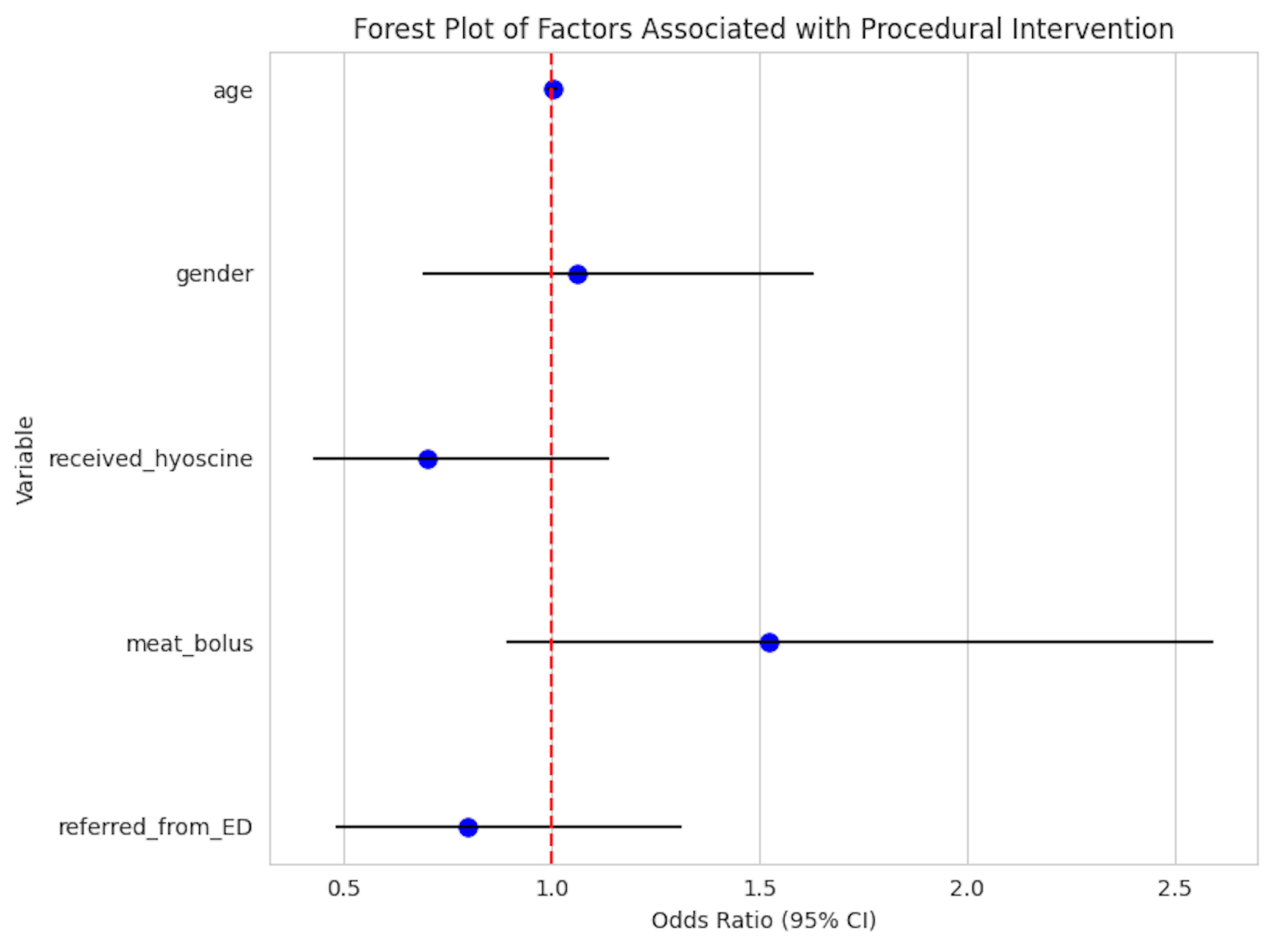
Forest plot of factors associated with the need for procedural intervention in OSFBO. This forest plot displays the adjusted odds ratios (OR) and 95% confidence intervals (CI) from multivariate logistic regression analysis identifying clinical and demographic factors associated with the requirement for procedural intervention in oesophageal soft food bolus obstruction (OSFBO). Variables included age, gender, use of hyoscine butylbromide, nature of food bolus, and referral source. Factors with OR >1 indicate increased likelihood of needing procedural management. Statistically significant associations (p < 0.05) are highlighted in bold. This plot provides insight into predictors of intervention necessity to guide clinical decision-making.

Patients managed conservatively, and those undergoing procedural intervention had varied outcomes in time to resolution (Figure 1). Multivariate logistic regression (Table 4) identified increasing age as a significant predictor of requiring procedural intervention (odds ratio (OR) = 1.02 per year, 95% confidence interval (CI) = 1.01-1.04; p = 0.01). The use of hyoscine butylbromide was strongly associated with increased odds of intervention (OR = 4.50, 95% CI = 1.90-10.50; p < 0.001). No statistically significant associations were found for gender, presentation within 24 hours, or type of food bolus (Figure 2).

**Table 4:** Logistic regression analysis of factors associated with the need for procedural intervention (n = 270). Model statistics: χ²(5) = 34.6, p < 0.001; Nagelkerke R² = 0.22.

Variable	Odds ratio (OR)	95% confidence interval (CI)	p-value
Age (per year increase)	1.02	1.01 – 1.04	0.01
Gender (female vs. male)	1.12	0.65 – 1.93	0.68
Use of hyoscine butylbromide	4.50	1.90 – 10.50	<0.001
Presentation ≤24 hours	0.85	0.45 – 1.60	0.61
Food bolus type (meat vs. other)	1.35	0.70 – 2.62	0.37

## Discussion

This large retrospective case series of 384 patients presenting with OSFBO to two UK tertiary centres provides valuable insights into the demographics, management strategies, and outcomes of this condition. Our demographic findings are consistent with previous literature, showing a higher incidence in older and male patients [14]. This likely reflects age-related dental issues such as edentulism, leading to inadequate mastication [15], and a higher prevalence of underlying oesophageal pathologies, including strictures and gastro-oesophageal reflux disease in this population [16,17]. The predominance of meat as the obstructing food bolus (83% of cases with known food type) aligns well with established studies [18].

Our data highlight the use and potential benefits of flexible nasendoscopy (transnasal oesophagoscopy) as a minimally invasive tool in managing OSFBO. This technique, usually employed for laryngeal and pharyngeal assessment, was successfully used to dislodge impacted food boluses in seven (2%) patients without complications. Although the success rate of flexible nasendoscopy as an intervention cannot be precisely quantified here due to incomplete data on unsuccessful attempts, its safety profile and avoidance of general anaesthesia make it an attractive first-line option for suitable patients. This supports previous smaller studies advocating its wider adoption in ENT practice [19].

Regarding procedural intervention, rigid oesophagoscopy was required in 18% and flexible oesophagoscopy in 13% of cases. The complication rate was low, with only one oesophageal tear (0.7% overall, 1.4% among those undergoing rigid oesophagoscopy) successfully managed conservatively. This is notably lower than the 7.2% complication rate reported in a meta-analysis comparing rigid and flexible oesophagoscopy for foreign body removal [8]. The lower risk in our series may be related to the exclusive focus on soft food bolus obstruction, which generally carries fewer risks than sharp or complex foreign bodies.

Our analysis of hyoscine butylbromide usage reveals that 71% (n = 193) of patients received at least one dose, but paradoxically, those who received hyoscine were more likely to require procedural intervention (32%, n = 62) compared to 7.8% (n = 6) in those not given the medication. The calculated odds ratio for requiring procedural intervention when treated with hyoscine was 5.3 (95% CI = 2.1-13.3), indicating significantly higher odds in this group. However, this association likely reflects selection bias: patients receiving hyoscine may have presented with more severe or prolonged obstruction, less likely to resolve spontaneously. This is consistent with prior observational studies showing no clear benefit of hyoscine butylbromide in OSFBO resolution [20,21] and the inconclusive results of randomized trials involving other pharmacological agents [2]. Given that 65% of patients resolved without procedural intervention overall, further prospective studies are warranted to clarify the role of pharmacological therapy.

The low diagnostic yield of neck and chest radiographs is also evident, with only 6% (n = 7) of X-rays showing signs of food bolus impaction. This supports current guidelines recommending radiology only when the nature of the obstruction is unclear or if a radiopaque foreign body is suspected [5]. Radiological imaging did not alter management in any cases in our series.

Contrast swallow radiography performed post resolution revealed oesophageal abnormalities in 48% (n = 36/75) of patients, predominantly achalasia and oesophageal webs (22% each). While previous studies have reported oesophageal strictures as the most frequent pathology in OSFBO [22,23], this discrepancy may arise from differences in diagnostic modality, as our study relied on contrast swallow rather than endoscopic visualization.

There are inherent limitations to this observational study. The absence of randomization and potential selection biases preclude definitive conclusions on treatment efficacy. Additionally, variations in referral pathways meant otolaryngology managed predominantly proximal obstructions (above the clavicles), typically treated by rigid oesophagoscopy, while gastroenterology managed more distal cases [24]. This anatomical heterogeneity was not analysed and may influence outcomes. Furthermore, data on biopsy findings, especially regarding eosinophilic oesophagitis, a condition increasingly recognized as an important underlying cause of OSFBO, were not captured [25].

Despite these limitations, this study provides comprehensive real-world data on OSFBO management, highlighting the safety and utility of flexible nasendoscopy, the limited role of radiological investigations, and the unclear benefit of hyoscine butylbromide. Our Kaplan-Meier analysis demonstrated that 82% of patients presented within 24 hours, emphasizing the acute nature of this condition. The forest plot illustrating the odds ratio of procedural intervention related to hyoscine use underscores the need for cautious interpretation due to confounding factors.

Future prospective studies, ideally randomized controlled trials, are needed to evaluate pharmacological interventions, including hyoscine butylbromide, and to optimize management protocols. In the meantime, ENT clinicians should consider early use of flexible transnasal oesophagoscopy to minimize the need for general anaesthesia and reduce complication rates. Additionally, post-resolution imaging with contrast swallow can identify underlying oesophageal pathology warranting further investigation or intervention.

## Conclusions

This large retrospective study confirms that the majority of OSFBO cases resolve spontaneously or with conservative management, underscoring the importance of careful patient selection and observation in the initial treatment approach. When procedural intervention is required, both rigid and flexible oesophagoscopy demonstrate high efficacy in resolving obstruction. However, rigid oesophagoscopy carries a notably increased risk of oesophageal trauma and perforation, highlighting the need for judicious use and consideration of less invasive alternatives when appropriate.

Importantly, post-resolution follow-up with contrast swallow radiography, though currently underutilized, reveals a substantial incidence of underlying oesophageal abnormalities, such as achalasia, webs, and strictures, that may predispose patients to recurrent obstruction or other complications. This emphasizes the critical role of contrast swallow imaging as a valuable diagnostic tool in the long-term management and risk stratification of patients after OSFBO episodes. Together, these findings advocate for a management paradigm that prioritizes conservative treatment and minimally invasive diagnostic and therapeutic techniques while incorporating systematic post-obstruction evaluation to identify and address underlying oesophageal pathology. Further prospective studies are warranted to optimize treatment algorithms and improve patient outcomes in this common but potentially serious clinical condition.
